# Adeno-Associated Virus (AAV)-Mediated Gene Therapy for Duchenne Muscular Dystrophy: The Issue of Transgene Persistence

**DOI:** 10.3389/fneur.2021.814174

**Published:** 2022-01-05

**Authors:** Arianna Manini, Elena Abati, Andi Nuredini, Stefania Corti, Giacomo Pietro Comi

**Affiliations:** ^1^Department of Pathophysiology and Transplantation (DEPT), University of Milan, Milan, Italy; ^2^Neurology Unit, Neuroscience Section, Dino Ferrari Center, Fondazione Istituto di Ricovero e Cura a Carattere Scientifico (IRCCS) Ospedale Maggiore Policlinico, Milan, Italy

**Keywords:** Duchenne muscular dystrophy, adeno-associated virus, gene therapy, persistence, dystrophin, microdystrophin

## Abstract

Duchenne muscular dystrophy (DMD) is an X-linked recessive, infancy-onset neuromuscular disorder characterized by progressive muscle weakness and atrophy, leading to delay of motor milestones, loss of autonomous ambulation, respiratory failure, cardiomyopathy, and premature death. DMD originates from mutations in the *DMD* gene that result in a complete absence of dystrophin. Dystrophin is a cytoskeletal protein which belongs to the dystrophin-associated protein complex, involved in cellular signaling and myofiber membrane stabilization. To date, the few available therapeutic options are aimed at lessening disease progression, but persistent loss of muscle tissue and function and premature death are unavoidable. In this scenario, one of the most promising therapeutic strategies for DMD is represented by adeno-associated virus (AAV)-mediated gene therapy. DMD gene therapy relies on the administration of exogenous micro-dystrophin, a miniature version of the dystrophin gene lacking unnecessary domains and encoding a truncated, but functional, dystrophin protein. Limited transgene persistence represents one of the most significant issues that jeopardize the translatability of DMD gene replacement strategies from the bench to the bedside. Here, we critically review preclinical and clinical studies of AAV-mediated gene therapy in DMD, focusing on long-term transgene persistence in transduced tissues, which can deeply affect effectiveness and sustainability of gene replacement in DMD. We also discuss the role played by the overactivation of the immune host system in limiting long-term expression of genetic material. In this perspective, further studies aimed at better elucidating the need for immune suppression in AAV-treated subjects are warranted in order to allow for life-long therapy in DMD patients.

## Introduction

Duchenne muscular dystrophy (DMD), the most common infancy-onset muscular dystrophy, is an X-linked recessive neuromuscular disorder characterized by progressive muscular weakness, wasting, muscle fatty infiltration, and fibrosis (1). By the age of 2–3 years, DMD patients develop lower limb weakness, leading to the delay of motor milestones, walking disturbances, frequent falls and, in the second decade of life, loss of autonomous ambulation (2). During disease progression, patients show respiratory failure, which requires ventilatory support, and cardiomyopathy, which represents the leading cause of death in DMD (3, 4). Almost one third of patients present neuropsychiatric disturbances, including intellectual disability, autistic spectrum disorders, hyperactivity and inattention (5).

DMD is caused by mutations in the *DMD* gene, which is composed of 79 exons and encodes for the dystrophin protein (6). DMD-causing mutations are mainly frameshift (deletions and, to a lesser extent, duplications, that generate a shift in the translational open reading frame (ORF) of the amino acid chain) or, in 13% of cases, non-sense (point mutations that replace a codon for an amino acid with a stop codon), thus resulting in a complete absence of dystrophin (7). Conversely, ORF-preserving mutations within the *DMD* gene produce a truncated but partially functional protein, leading to the development of variable phenotypes, ranging from an asymptomatic state to Becker muscular dystrophy (BMD) or DMD (8, 9).

Dystrophin is a 427 kDa cytoskeletal protein composed of four domains: the N-terminal domain; the rod domain, containing 24 triple helix spectrin-like repeats and four hinges; the cysteine-rich domain; the C-terminal domain (10). Together with sarcoglycans, dystroglycans, sarcospan, syntrophin, dystrobrevin, and neuronal nitric oxide synthase, it belongs to the dystrophin-associated protein complex (DAPC) (11), which is involved in cellular signaling and plays a crucial role in the stabilization of myofiber membrane (11). Dystrophin loss results in enhanced vulnerability of the sarcolemma and consequent induction of myofiber split during contraction, functional ischemia, free radicals-mediated oxidative damage, and overload of cytosolic calcium, ultimately leading to mitochondrial dysfunction and myofiber death (12–16). Furthermore, the regenerative ability of myofibers in DMD is impaired, with consequent fibrotic replacement of muscle tissue (17).

To date, no disease-modifying therapeutic option is available for DMD. Current treatments are aimed at lessening disease progression, but premature death is unavoidable. Guidelines for optimal care include multidisciplinary medical, surgical, and rehabilitative strategies, including annual monitoring of respiratory and cardiac function from diagnosis (to be increased after the loss of ambulation and the onset of cardiac symptoms, respectively), the use of mechanically assisted coughing and ventilation devices in case of hypoventilation, and treatment with angiotensin-converting enzyme (ACE) inhibitors by the age of 10 years (1). Corticosteroids are part of the current standard of care (1). Although there is no clear consensus on the timing of glucocorticoid initiation, prednisone or deflazacort are usually introduced after the onset of motor function decline and continued throughout life, as they have been shown to improve muscle force and function, to delay cardiac and respiratory impairment and loss of ambulation, and to reduce the need of surgery for scoliosis (1). The optimal regimen is controversial, as available data from randomized controlled trials are not sufficient to infer conclusions on long-term efficacy and adverse effects of daily vs. intermittent treatment (18).

**Figure 1:**
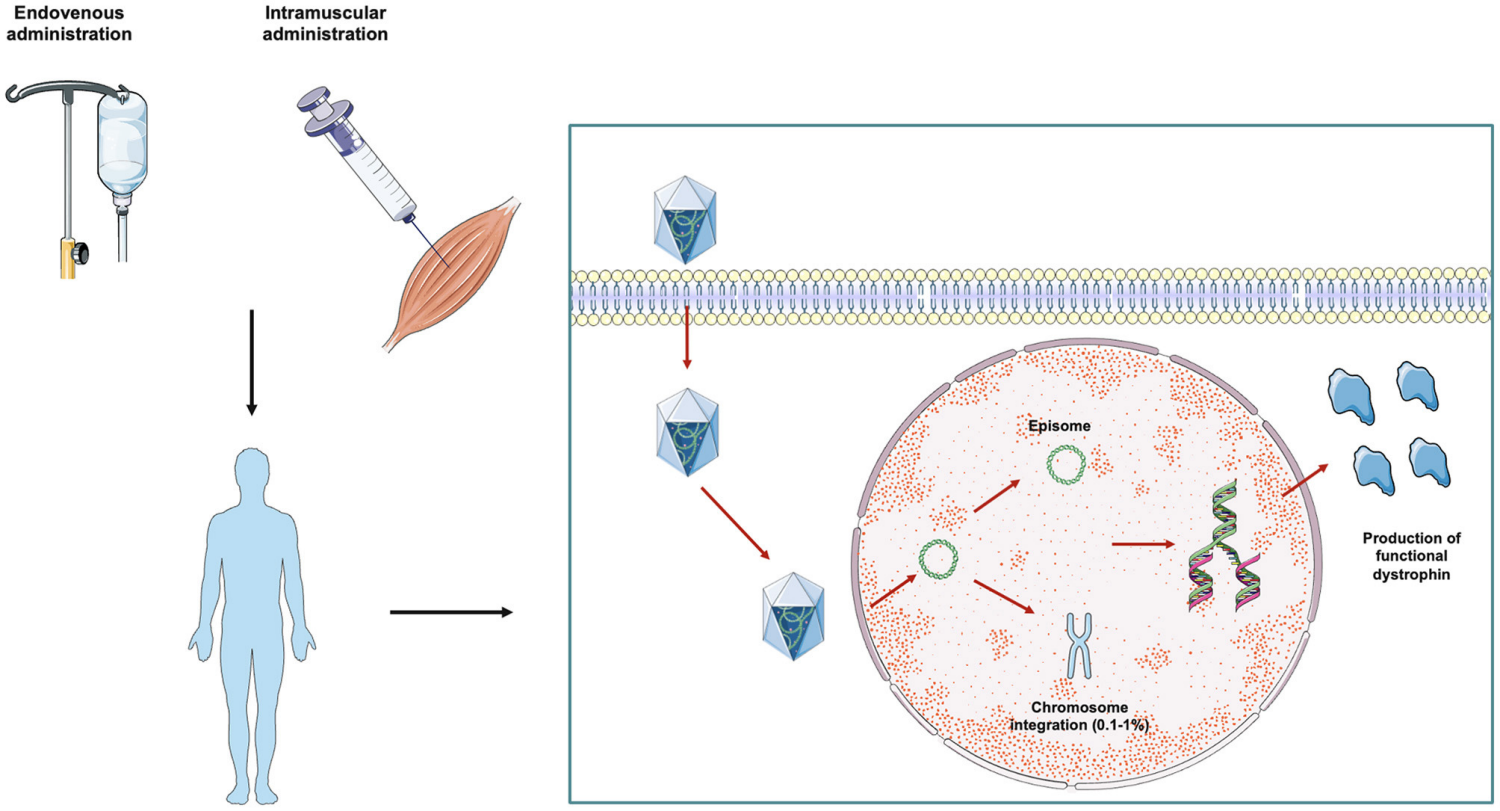
AAV-mediated gene therapy in DMD from bench to bedside. Gene therapy approaches in DMD currently under study are based on the delivery of reduced-length *mini-* or *micro-dystrophin* protein packaged within an adeno-associated virus (AAV). The therapeutic vector can be administered systemically via the intravenous route or directly within the involved muscles. After the injection, AAV vectors are imported inside the cell by internalization in clathrin coated endocytic vesicles. The vectors are then released from the vesicles into the cytoplasm and translocated to the nucleus where transgenes are released. Once inside the nucleus, the exogenous DNA particle remains in transduced cells in episomal state, and only a very small percentage (0.1–1%) of the vector genome is integrated into the host chromosome. Transduced cells then start expressing the transgene, with consequent production of a truncated, but functional, dystrophin protein.

In this scenario, one of the most promising therapeutic strategies for DMD is represented by gene therapy (19) (Figure 1). Gene therapy aims at the restoration of the deficient protein by delivering an exogenous, functional gene to target tissues with the aid of recombinant adeno-associated viruses (rAAVs). The aim of gene replacement therapy is to provide a therapeutic effect by addressing the genetic root of the disease and improving or reverting disease phenotype. The first gene therapies were approved for human use by the Food and Drug Administration (FDA) in 2017, leading to commercialization of Spark Therapeutics' Luxturna for the treatment of RPE65 mutation-induced blindness and Novartis' Kymriah, the first chimeric antigen receptor T cell therapy (20, 21). Since then, several other gene therapies have been approved. In the neuromuscular field, the approval of gene therapy for Spinal Muscular Atrophy (SMA), aimed at replacing the *SMN* gene, represented a landmark event (22). Several other gene therapy strategies for treating other neuromuscular diseases, including Duchenne's Muscular Dystrophy, are now under development. The success of therapy is contingent on directing a gene to the correct cells. While non-viral delivery models are under investigation, the most common approach is via viral vectors.

Here, we summarize preclinical and clinical studies of AAV-mediated gene therapy in DMD, as well as the main features of AAV biology. We specifically focus on the concept of transgene persistence in cells, with the aim of unraveling potential obstacles in the maintenance of long-term therapeutic effects of gene therapy.

## Adeno-Associated Viruses for Gene Therapy

As mentioned above, a considerable issue regarding molecular therapy is optimal delivery, as gene therapy-based strategies require delivery tools that can enable maximal intracellular entry (23). Given their small dimensions, high lipophilicity and reduced binding to serum proteins, molecules such as polymeric nanoparticles (NPs) and lipid complexes might serve this function (24). Nevertheless, viral vectors, in particular retroviruses, lentiviruses, adenoviruses, and AAVs, which are able to infect host cells and replicate through the host machinery (and are thus able to cross the BBB and easily reach target sites), are the most commonly used tools.

The selection of a specific viral vector for gene therapy is based on several factors. First, its specific tropism, made possible by the tight interaction between host cells and vector receptors, with highly efficient transduction to target cells and limited diffusion to other tissues. In addition to that, the need to keep the number of the potential side effects, usually associated with host immune response, at a minimum. Another factor to consider is the long-term expression of the transgene. Ideally, a single dose of AAV-mediated gene therapy should achieve therapeutic protein levels with no toxic effects. Moreover, a non-integrating viral vector is preferable to maintain the transgene in the form of extrachromosomal episome, thus reducing the risk of pro-oncogenic mutagenic effects. Current efforts are directed at the production of the optimal viral vector, with the aid of engineering techniques aimed at overcoming either packaging limitations, with the introduction of novel serotypes or modification of natural capsids, and host immune responses against the vector itself.

In the field of neurologic diseases, the most widely used and studied vectors are AAV vectors, as discussed in the following paragraphs.

### Adeno-Associated Virus Biology

Discovered in 1965, theAAVs (Parvoviridae family, Dependovirus genus), are single-stranded DNA viruses with a small (20–28 nm) protein capsule (24). Dependoviruses are characterized by defective replication, requiring a co-infection with an adenovirus or herpesvirus for productive replication in mammalian cells (25).

The 4.7 kb single stranded linear genome can be both plus (sense) or minus (antisense) and contains four ORF: the *rep*, responsible for the coding of four replication proteins, termed according to their molecular weight (Rep78, Rep68, Rep52, and Rep40); the *cap*, which encodes the three capsid (Cap) proteins VP1, VP2, and VP3; the *aap*; the recently identified *map* (26, 27). The genome is flanked on 5′ and 3′ end by inverted terminal repeats (ITR) made of 145 nucleotides. Genome replication starts from the first 125 ITR nucleotides, which form a T-shaped stem loop structure, and proceeds through a rolling hairpin mechanism (28, 29).

Rep78 and Rep68 cut the terminal AAV site and bind terminal hairpin DNA substrates to process terminal resolution, reinitiation, and strand displacement during AAV replication (30). Rep52 and Rep40 are involved in the accumulation of viral genome and packaging within AAV capsid (30, 31). Rep 78 and 68 can bind the viral genome to a 6-nucleotides recognition sequence (AAVS1 locus) at the human chromosome 19q13. If cellular conditions cannot allow viral replication, the Rep proteins repress the p5 promoter and the viral genome is integrated at the AAVS1 locus, thus guaranteeing a long-term expression both in dividing and non-dividing cells (32). Integration of viral genome into the host genome is, however, a rare phenomenon in mammalian tissues, particularly in skeletal and cardiac muscles (33).

In the recombinant AAV (rAAV), viral genes are replaced by the transgene (34, 35). To allow the packaging of the transgene into the viral capsid, the transgene cassette is flanked by the ITR sequence (34). As rAAVs lack the *rep* gene, integration difficulty occurs, and with no specificity for the AAVS1 locus (36).

Based on capsid proteins, the AAVs are classified in more than 10 serotypes, characterized by different cell tropism (37–47), whose main features are summarized in Table 1. Several AAV serotypes, including rAAV1, rAAV2, rAAV5, rAAV6, rAAV7, rAAV8, and rAAV9, have shown efficient muscle transduction (37–39, 49, 61–64). As AAV8 and AAV9 can efficiently reach multiple muscles, they are ideal for widespread muscle disorders involving hearth and diaphragm, which can hardly be reached with local injections, as it happens in DMD. Furthermore, the transgene expression after AAV9-mediated cardiac fibers transduction is significantly higher than serotypes 4, 6, 7, and 8 (40, 41).

**Table 1:** List of AAV serotypes used for gene therapy in neuromuscular disorders.

**Serotype**	**Glycan recognition**	**Coreceptor**	**Target tissues**	**Source**	**Human neuromuscular diseases**	**Clinical trials**	**Ref**.
AAV1	Neu5Acα2-3GalNAcβ1-4GlcNAc	NA	Muscle, liver, CNS	Contaminant in simian adenovirus 15 stock	DMD, BMD, LGMD. ALS	NCT02354781, NCT00494195, NCT01344798	(41, 48–50)
AAV2	6-O- and N-sulfated heparin	Fibroblast/hepatocyte growth factor receptor; laminin receptor; integrin αVβ5 and α5β1	CNS, liver	Contaminant in adenovirus type 12 stock	LGMD	NA	(51–55)
AAV3	2-O- and N-sulfated heparin	Hepatocyte growth factor receptor; Laminin receptor	Liver	Contaminant in adenovirus type 7 stock	NA	NA	(53, 56)
AAV4	Galβ1-4GlcNAcβ1-2Manα1-6Manβ1-4GlcNAcβ1-4GlcNAc	NA	Lung, ependymal cells	African green monkeys infected with SV15	NA	NA	(48)
AAV5	Neu5Acα2-3(6S)Galβ1-4GlcNAc	Platelet-derived growth factor receptor	Muscle, liver, CNS, retina	Human penile condylomatous wat	DMD	NA	(48, 57)
AAV6	Neu5Acα2-3GalNAcβ1-4GlcNAc; N-sulfated heparin	Epidermal growth factor receptor	Muscle, liver, spinal cord	Contaminant in lab adenoviral stock	NA	NA	(48, 58)
AAV7	NA	NA	Muscle, liver	Rhesus monkeys	NA	NA	(48)
AAV8	NA	Laminin receptor	Muscle, liver, retina, pancreas	Rhesus monkeys	XLMTM	NCT03199469	(48, 53)
AAV9	Galactose	Laminin receptor	CNS, skeletal and cardiac muscles, liver	Human	SMA, DMD	NCT03505099 (SPR1NT, Phase III); NCT03461289 (STRIVE-EU, Phase III); NCT03306277 (STR1VE, Phase III) NCT03362502, NCT03368742	(39, 53, 59)
AAVrh.74	NA	NA	Muscles, spleen, liver	Rhesus macaque mesenteric lymph nodes and spleen	DMD and with limb-girdle muscular dystrophy types 2E/R4, 2D/R3, and 2B/R2	NCT02376816, NCT03769116, NCT03652259, NCT01976091, NCT02710500	(60)

*CNS, central nervous system; NA, not available; DMD, Duchenne muscular dystrophy; BMD, Becker muscular dystrophy; LGMD, limb girdle muscular dystrophy; ALS, amyotrophic lateral sclerosis; SMA, spinal muscular atrophy; XLMTM, X-linked myotubular myopathy*.

### Molecular Bases of Transgene Persistence

Preclinical studies have largely demonstrated that the rAAV vector persists *in vivo* as head-to-tail concatemers mostly organized in episomes (42). The single-stranded rAAV genome is released in the cellular nucleus where the second strand synthesis starts from the 3′-end (43). Subsequently, the double strand genome circularizes and forms high-molecular-weight concatemers (43). These circular intermediates have shown sustained persistence in muscular cells as well as increased episomal stability following liposome-mediated transfection (44). By injecting a shuttle virus, termed V.GFP3ori, containing a green fluorescence protein (GFP) reporter gene in the tibialis anterior muscle of mice, Duan and colleagues demonstrated the presence of circular intermediates with a characteristic head-to-tail structure with a peak of the GFP transgene expression at 22 days, which remained stable for at least 80 days (44). While circular intermediates initially occur as monomer head-to-tail genomes, at 80 days most of them recombine into larger multimers and persist in the episomal state (44). This was suggested to be the principal source of long-term expression, as circular conformation may have an increased resistance to nucleases (44). Furthermore, these circular intermediates showed two ITRs in a head-to-tail orientation, which significantly increased the stability of DNA following transfection into HeLa cells (44).

In dividing cells, persistence is deeply dependent on division rate. For instance, in hepatic cells, which show the highest rate of integration within mammalian cells (<1%), up to 90% of the rAAV genome is loss after surgical hepatectomy, probably because of the loss of episomes with cell division, which is enhanced by the surgical procedure (45). Conversely, in mice that did not undergo hepatectomy, a significant number of rAAV genomes remained non-integrated, leading to persistent transgene expression for up to 19 months (45). A sustained and prolonged (>17 months) expression of the transgene was demonstrated also in a canine model of hemophilia B after a single intramuscular (IM) injection of AAV carrying the transgenic factor IX (46).

Recently, efforts have been made to engineer vectors that could persist in the episomal form in dividing cells, thus allowing a long-term expression with a very low risk of genotoxicity associated with insertional mutagenesis. One approach is based on AAV vectors with a scaffold/matrix attachment region (S/MAR) which maintains long-term transgene expression (>50 population doublings) by replicating AAV vector episomes in the absence of further selection (47). Alternatively, somatic integration of the AAV vector genome into the host chromosome can be enhanced via the Sleeping Beauty (SB) transposase integration machinery (65). This system consists of one AAV vector carrying the transposon with the target gene, and a second one which delivers the hyperactive SB100X transposase (65).

As transgene expression depends on second-strand synthesis, the speed and rate of cell transduction is limited. To overcome this limitation, self-complementary (sc) AAV vectors were developed. Compared to double stranded (ds) and single stranded (ss) AAV, the efficiency of cell transduction of scAAV vectors was significantly higher (66, 67), mainly due to the ability of scAAV to circumvent the step of second strand DNA synthesis mediated by the host cell (68).

### Engineering Strategies to Increase Transduction Efficiency

The use of rAAVs with natural capsid variants, which are seized in the liver after systemic injection, requires elevated virus doses to reach therapeutic threshold in muscles, thus enhancing therapy-related toxicity (69, 70). Therefore, the DELIVER (directed evolution of AAV capsids leveraging *in vivo* expression of transgene RNA) strategy was recently applied to engineer AAVs with common arginine-glycine-aspartic acid (RGD) motif-containing capsid variants (MyoAAV 1A) showing higher potency and transduction selectivity of both skeletal and cardiac muscles in mice after systemic and IM administration compared to AAV9 (71). Although MyoAAV 1A was associated with a reduced transduction of liver compared to AAV9, levels of liver enzymes in the serum of AAV9 and MyoAAV1A-injected mice did not differ from controls, thus suggesting absence of hepatic injury at injected doses (71). Using both a CRISPR-Cas9-mediated approach and gene replacement, the authors demonstrated that MyoAAV 1A-mediated systemic delivery of therapeutic transgenes resulted in higher widespread dystrophin rescue in muscles and muscle functional improvement (i.e., greater specific force, lower force drop after eccentric contraction) in mice compared to AAV9 (71). As the effect of MyoAAV 1A on myotubes transduction was mediated by the interaction between RGD motifs and integrin heterodimers, the authors modified amino acids adjacent to the RGD motif to engineer second-generation AAV (MyoAAV 2A) with enhanced muscle tropism, which showed greater muscle transduction efficiency in mice and human primary myotubes compared to AAV9 and MyoAAV 1A (71). As MyoAAV 1A, MyoAAV2-mediated gene therapy in mice led to increased dystrophin rescue and muscle functional improvement (71). Similarly, MyoAAV 4A, 4E, 3A, and 4C were generated and demonstrated higher transduction of skeletal muscle potency in NHPs compared to AAV9 and AAVrh74 (71), which is currently used in DMD gene therapy clinical trials (60).

The advantages of AAV vectors are the lack of transfection-related diseases, the ability to transduce both dividing and non-dividing cells, the reduced immunogenicity and the long-term expression of the delivered transgene in specific tissues (35). However, host immune responses, specifically neutralizing antibodies (NAbs), significantly limit AAV transduction capability *in vivo*. Noteworthy, up to 70% of the population was shown to carry a protective humoral immune response against AAVs, due to previous exposure to the viruses, with differences in the serotypes based on demographic features. Many attempts to escape host immune responses have been made, including injecting empty capsids as decoys, plasmapheresis and engineering rAAV capsids without Nab integrating epitopes (72–74). Another approach currently under investigation is the use of non-human derived capsids (75). In particular, capsids isolated from non-human primates (NHP) could be an option as NHP-derived AAVrh.8, AAVrh.10, and AAVrh.43 can efficiently transduce different tissues (75). Unfortunately, these approaches were not able to efficiently escape host immune responses. Therefore, individuals exhibiting NAbs are excluded *a priori* from clinical trials, as gene therapy is expected to be inefficient (27, 76).

### AAV-Related Serious Adverse Events in Human Neuromuscular Disorders

Within the most relevant issues related to AAV-mediated gene therapy lies the limited knowledge concerning possible serious adverse events (SAE), which represents a profound obstacle in moving gene therapy strategies to a clinical setting. Little is known, for instance, about AAV-related liver toxicity and its pathogenesis. In clinical trials enrolling spinal muscular atrophy (SMA) and DMD patients, a significant elevation of liver enzymes was described, although it was controlled with corticosteroids with no relevant clinical impact (39, 60, 77). However, in June 2021 Audentes Therapeutics reported the death of three patients affected by X-linked myotubular myopathy (XLMTM), who received the highest AAV dose ever used in humans (3 x 10^14^ vg/kg) in the context of the ASPIRO clinical trial (NCT03199469), aimed at evaluating safety and efficacy of AT132, an AAV8-delivered gene therapy (AAV8-MTM1) (69, 78). XLMTM is a lethal heterogeneous myopathy commonly fatal within 12 years of age, that results from mutations in myotubularin-encoding gene *MTM1* (79). From 4 to 6 weeks after administration, the two patients, who both had a history of hepatobiliary disease, underwent progressive liver dysfunction and sepsis leading to death (69). The timing, dose-dependency and tissue-specificity pointed toward an adaptive immune response against vector capsid or transgene product as primary cause of the recorded SAEs, although a definite etiology has not been proved yet (69).

In a phase Ib clinical trial involving 9 DMD patients who received a single intravenous administration of PF-06939926 (NCT03362502), preliminary data disclosed three SAEs, namely vomiting resulting in dehydration, acute kidney injury, and thrombocytopenia with atypical hemolytic uremic syndrome-like complement activation (80). All these SAEs resolved within 2 weeks, but their occurrence suggests that an overactive immune response toward the vector may hinder the application of gene therapy, at least at high doses (80).

## AAV-Mediated Gene Therapy in DMD

### Gene Replacement

The *dystrophin* gene has a coding sequence of 14 Kb, which largely exceeds the packaging size of AAV vectors. This has precluded the use of AAV-mediated gene replacement in DMD until miniature versions of dystrophin genes were developed. These micro (four or fewer repeats) and mini (more than four repeats) dystrophin genes encode for truncated but functional proteins lacking unnecessary domains, including a major portion of the rod domain and the distal C terminus domain (81, 82). The result is a milder dystrophic phenotype compared to untreated subjects (83).

AAV-mediated gene replacement was first tested in the *mdx* mouse model (84–90). The *mdx* mouse harbors a non-sense mutation in the exon 23 of the *Dmd* gene, which impairs dystrophin expression (91). After injecting several mini-dystrophin genes packaged into AAV vectors in the hindleg muscle (i.e., tibialis anterior) of both 10-day-old and adult *mdx* mice, Wang and colleagues demonstrated widespread expression of mini-dystrophins in most myofibers and significant reduction of pathological dystrophic features (85). Two years later, the same group repeated injections of mini-dystrophin genes packaged into AAV vectors in the hindleg muscle of adult *mdx* mice, revealing a significant improvement in muscle contractile function and resistance to mechanical stress (84). However, muscle strength of AAV-treated *mdx* mice remained significantly lower than the strength of control C57/B10 mice (84). Possible explanations are the low transduction efficiency of AAV serotype 1 in fast-twitch myofibers, which are predominant in the tibialis anterior muscle, or the reduced ability of mini-dystrophins to restore muscle force compared to full-length dystrophin (84).

As the *mdx* mouse shows a compensatory over-expression of dystrophin-related proteins such as utrophin, thus resulting in a milder dystrophic phenotype compared to DMD patients, Gregorevic and colleagues selected the dystrophin/utrophin double-knockout (*dko*) mouse model to test the intravascular administration of micro-dystrophin packaged into an AAV-6 (86). The authors showed widespread expression of dystrophin after 18 weeks in the diaphragm and cardiac muscle (86). In the diaphragm, they also demonstrated reduction of histological biomarkers of muscle dystrophy, increased muscle strength, and resistance to contraction-induced damage. The results obtained in skeletal muscles were similar to those reported before (86).

Promising results were obtained also in dog models of canine X-linked muscular dystrophy (CXMD), which were intramuscularly injected after immunosuppression with a combination of anti-thymocyte globulin (ATG), cyclosporine (CSP), and mycophenolate mofetil (MMF), so to guarantee both a long-term expression of the micro-dystrophin transgene and the restoration of DAPC at the sarcolemma (92). Indeed, previous studies reported the development of a T cell-mediated immune response after IM injection of AAV2 and AAV6, which compromised sustained AAV-mediated dystrophin expression in models other than mice (93). After discontinuation of CSP and MMF, patches of T-cell infiltration were shown (92). However, the expression of micro-dystrophin persisted in areas with reduced or absent T-cell infiltrates (92).

Although AAV9 apparently avoided the development of an immune response when intravenously injected in neonatal dogs in a previous study, Kornegay and colleagues could not replicate the aforementioned promising results in the golden retriever muscular dystrophy (GRMD) model, intravenously injected with an AAV9 vector carrying a human mini-dystrophin in absence of immunosuppression (64, 94). Even though mini-dystrophin was still expressed in almost all myofibers of different muscles at 16 weeks after treatment, treated GRMD dogs showed pelvic limb muscle atrophy and contractures, and mild degeneration and regeneration features of myofibers at histological examination, accompanied by severe fatty replacement (64). Conversely, immunosuppression in juvenile DMD dogs intravenously injected with a micro-dystrophin AAV9 vector variant, with a surface tyrosine mutated to reduce immunogenicity, allowed not only a widespread, long-term muscle transduction in skeletal muscle, diaphragm and heart, but also improvement in muscle histology (95).

Intriguingly, Le Guiner and colleagues reached long-term (up to 24 months) high levels of micro-dystrophin expression in skeletal muscles and marked improvement of histological and functional parameters after intravenous delivery of recombinant AAV2/8 vector expressing a canine micro-dystrophin in GRMD dogs, in absence of immunosuppression (96). Possible explanations of the lack of detectable cellular immune response resulting in long-lasting transgene expression might include the use of a muscle-specific synthetic promoter, and factors related to the recombinant AAV2/8 production protocols (96).

Before moving to the human setting, Rodino-Klapac and colleagues tested the feasibility and efficacy of a FLAG-tagged (to differentiate endogenous and vector-derived protein) micro-dystrophin, packaged into an AAV8 vector, and delivered in rhesus macaques via IM and intraarterial route (97). The authors observed a strong and long-term (at least 3–5 months) gene expression (50–79% of myofibers after IM injection in the tibialis anterior muscle; >80% of myofibers of the gastrocnemius muscle after intra-arterial injection in the femoral artery of rhesus macaques without pre-existing AAV8 antibodies), without evidence of immune cellular response (97).

The first randomized double-blind placebo-controlled phase I clinical trial aimed at assessing the impact of the IM delivery of an AAV micro-dystrophin gene (rAAV2.5-CMV-mini-dystrophin, d3990) (NCT00428935) failed to confirm the promising results obtained in animal models (98, 99). Six patients with frame-shifting deletions in the dystrophin gene were enrolled and followed up between 2006 and 2009, and received two different doses of vector genome (2.0 x 10^10^/kg and 1.0 x 10^11^/kg) via injection in the biceps muscle (98, 99). To enhance the muscle transduction capacity of the adenoviral vector, the AAV2.5 was generated from the AAV2 capsid by adding five mutations from AAV1 (99). Quantitative polymerase chain reaction (PCR) confirmed the presence of the transgene DNA in both injected and contralateral arms of all patients except one, in whom spread to the contralateral arm was not found (99). Possible explanations of the minimal long-term gene expression (0/6 patients at 90 days; 2/6 patients at 42 days) include the development of a T cell-mediated immune response, pre-existing anti-AAV antibodies (detected in 2/6 patients), pre-existing T cells targeting endogenous revertant dystrophin epitopes which are shared by the mini-dystrophin employed (detected in 2/6 patients), dystrophy-related inflammation, reduced tropism of AAV2.5 to human muscles and silencing of the CMV promoter in human dystrophic myofibers (98, 99).

In September 2020, Mendell and colleagues published the results of an open-label, phase I/IIa, non-randomized, controlled trial (NCT03375164), which enrolled 4 ambulatory DMD patients (mean age at enrollment, 4.8 years), carrying a confirmed *DMD* frameshift or premature stop codon mutation between exons 18 and 58, without preexisting AAVrh74 antibodies, who had been on a stable corticosteroid dose for at least 12 weeks before entry (60). Patients received a single dose of intravenous 2.0 × 10^14^ vg/kg rAAVrh74.MHCK7.micro-dystrophin (SRP-9001), together with prednisone at high doses for 30 days, followed by a slow taper (60). The MHCK7 promoter was selected for its high levels of expression in both skeletal muscles, including the diaphragm, and hearth (100). The micro-dystrophin was well-tolerated, with only mild-to-moderate adverse effects (*n* = 53); only 18 of them were considered treatment-related, the most common being vomiting (*n* = 9) (60). Immunohistochemistry of gastrocnemius muscle biopsy performed at 12 weeks showed strong transgene expression, and improvements both in the CK levels and in the North Star Ambulatory Assessment (NSAA) score were demonstrated after 1 year (60).

In May 2020, the preliminary data of a phase Ib clinical trial conducted on 9 ambulatory DMD patients, who received a single intravenous administration of PF-06939926 (NCT03362502) and were followed up for 12 months, were presented at the American Society of Gene and Cell Therapy (ASGCT) Annual Meeting (80). Moreover, robust expression of micro-dystrophin persisted at 12 months, together with improvement in the NSAA score (80).

Solid Biosciences have recently reported interim efficacy and safety encouraging data from the ongoing phase I/II IGNITE DMD Clinical Trial (NCT03368742) from six patients treated with either a low or high dose of SGT-001, a micro-dystrophin characterized by the addition of a neuronal nitric oxide synthase (nNOS)-binding domain to the cassette to enhance muscle blood flow, thus supporting the continued enrollment of patients (101).

As summarized in Table 2, several clinical trials of AAV-mediated gene replacement are ongoing (NCT03375164; NCT03368742) or have been completed (NCT02376816), although preliminary results have not been published yet.

**Table 2:** Clinical trials of AAV-mediated gene therapy in DMD.

**Intervention**	**Clinical trial phase**	**Patients enrolled**	**AAV serotype**	**Delivery route**	**Primary outcome**	**Primary outcome measure data**	**Micro-dystrophin expression**	**Current stage**	**Clinical trial number**	**Ref**
rAAV2.5-CMV-minidystrophin (d3990)	I	6	rAAV2.5	Intramuscular (biceps muscle)	Adverse events	Nausea or upset stomach 1/6 Macular rash 2/6 Fungal rash 1/6 Sore throat 2/6	0/6 at 90 days 2/6 at 42 days	Completed	NCT00428935	(98, 99)
rAAVrh74.MCK.micro-dystrophin	I	2	rAAVrh74	Intramuscular (extensor digitorum brevis)	Adverse events	NA	NA	Completed	NCT02376816	NA
PF-06939926	Ib	NA (estimated 30)	rAAV9	Intravenous	Adverse events	NA	NA	Active, not recruiting	NCT03362502	NA
PF-06939926	III	NA (estimated 99)	rAAV9	Intravenous	NSAA	NA	NA	Recruiting	NCT04281485	NA
SGT-001	I/II	NA (estimate 16)	rAAV9	Intravenous	Adverse events, abnormalities in laboratory findings, vital signs, physical examination, ECGs, micro-dystrophin Expression in muscle biopsy	NA	NA	Recruiting	NCT03368742	NA
rAAVrh74.MHCK7. micro-dystrophin (SRP-9001)	I/IIa	Actual 4 (estimate 12)	rAAVrh74	Intravenous	Adverse events	Vomiting 14/53 Fecal incontinence 1/53 GERD 1/53 Loose stool 1/53 Nausea 1/53 Abnormal GI distension 1/53 URI 9/53 Viral illness 2/53 Viral gastroenteritis 1/53 Pain at biopsy site 3/53 Fatigue 2/53 Asthenia 1/53 Fever 1/53 Foot pain 1/53 Decreased appetite 3/53 Hypokalemia 1/53 Elevated liver enzymes 4/53 Asthma exacerbation 1/53 Cough 1/53 Proteinuria 1/53 Eye irritation 1/53 Headache 1/53	4/4 at 12 weeks	Active, recruiting	NCT03375164	(60)
rAAV1.CMV. huFollistatin344	I/II	3	rAAV1	Intramuscolar (gluteal, quadriceps and tibialis anterior muscles)	Adverse events 6MWT	Head injury 1/3 Stye 1/3 GERD 1/3 Constipation 1/3 Influenza 1/3 Pain 3/3 Compression fracture 1/3 Increased muscle weakness 1/3 Anxiety 1/3 Behavioral changes/agitation 1/3 Insomnia 1/3 Pharyngitis /3 Rhinorrhea 1/3 Nasal congestion 1/3 Abrasion 1/3 Bruising 2/3	NA	Completed	NCT02354781	NA
rAAVrh74.MCK. GALGT2	I/IIa	NA (estimate 6)	rAAVrh74	Intra-arterial	Unacceptable toxicity	NA	NA	Active, not recruiting	NCT03333590	NA
scAAV9.U7.ACCA	I/II	3	scAAV9	Intravenous	Unacceptable toxicity	NA	NA	Enrolling by invitation	NCT04240314	NA

*r, recombinant; AAV, adeno-associated viruses; CMV, Cytomegalovirus; MCK, muscle creatine kinase; NA, not available; MHCK, myosin heavy chain kinase; 6MWT, 6 Minute Walk Test; GERD, gastroesophageal reflux disease; URI, upper respiratory tract infection; GI, gastrointestinal; sc, self-complementary; NSAA, North Star Ambulatory Assessment; Ref, reference*.

### Gene Replacement Surrogates

Utrophin is a cytoskeletal protein located at the neuromuscular and myotendinous junctions (102). It represents an autosomal paralog of dystrophin with about 80% homology, and its AAV-mediated over-expression has been investigated in several DMD animal models (103–105). Indeed, the AAV-mediated gene transfer of a shortened version of utrophin, delivered by IM injection in newborn *mdx* mice, was able to restore the DAPC at the sarcolemma, to reduce histological dystrophic features (i.e., central nucleation), to protect the sarcolemma from injury, and to enhance muscle strength and resistance to mechanical stress-induced damage (103). These findings were not observed in 30–45-day-old mice after IM injection, because of the development of a humoral response (103). Subsequently, a micro-utrophin packaged into a recombinant AAV6 vector was intravenously delivered in 1 month-old *mdx/Utr*^+/−^ mice, which have a more severe phenotype compared to *mdx* mice, thus mimicking human DMD patients (104). Micro-utrophin was subsequently detected not only at the myotendinous junction, but also within the sarcolemma, unlike matched wild-type control mice (104). Consistent with previous results, the AAV-mediated micro-utrophin over-expression rescued the DAPC components at the sarcolemma. Compared with controls, the treated *mdx/Utr*^+/−^ mice showed increased body and heart mass, reduced CK levels, mitigation of histological dystrophic features (i.e., inflammatory infiltrates, central nucleation), and significant increase in muscle force generation capacity (103, 104). More recently, the systemic AAV-mediated delivery of artificial zinc finger transcription factors (ZF-ATFs) targeting the utrophin A promoter have been tested in *mdx* mice with the aim of up-regulating the utrophin expression levels (106, 107). The authors described a significant reduction in muscle pathological and biochemical features (i.e., inflammatory infiltrates, central nucleation, CK levels), an improvement in exercise performance and contractile activity, and an enhanced acetylcholine receptors postsynaptic clustering (107).

The β1-4-N-acetyl-D-galactosamine (βGalNAc) glycosyltransferase, encoded by the *GALGT2* gene, is involved in glycosylation of α-dystroglycan in myofibers (108). The AAV-mediated delivery of GALGT2 via femoral artery in the rhesus macaque allowed a significant gene expression (44%) in myofibers, which was reduced to 9% in presence of pre-existing antibodies, with increased glycosylation of α-dystroglycan and expression of dystrophin surrogate proteins (i.e., utrophin) (109). The rAAVrh74.MCK.GALGT2 is currently being tested in a phase I/II clinical trial (NCT03333590), aimed at assessing the safety of intravascular lower limb infusion in 6 DMD patients (Table 2).

Follistatin (rAAV1.CMV.huFollistatin344), a potent myostatin inhibitor, obtained encouraging results in pre-clinical setting and in BMD patients, and is currently being tested in a phase I/II clinical trial (NCT02354781) (50, 110). Three DMD patients have received the drug via IM injection in the gluteal muscles, quadriceps, and tibialis anterior of 3 DMD patients. The primary endpoint is the overall improvement in the 6 Minute Walk Test (6MWT), but related results have not been published yet (Table 2). Among the adverse events recorded so far, only one was deemed serious, namely head injury from fall.

## Persistence of AAV Genome Within Myofibers and Cardiac Fibers After Gene Replacement

One of the biggest issues regarding effectiveness and sustainability of gene therapy for DMD is the uncertainty related to its long-term persistence. Indeed, as muscular tissue is capable of proliferation and renovation, and the cycle of degeneration and regeneration is particularly active in DMD muscles, it is expected that the expression of transgene could fade over time. To this regard, results obtained in healthy muscle tissue, as well as those arising from administration of transgenes in non-muscle tissues, hold limited value. To date, available studies have not yielded consistent results as regards persistence of DMD-targeting transgenes within muscle tissues, nor they were able to undoubtedly point out optimal timing and route of administration to improve long term expression. The limited life-span of animal models, especially mice (2–3 years), represents a significant challenge for preclinical studies focused on persistence of transgene expression after gene replacement, as it intrinsically cannot be employed to investigate the longer duration of expression required by DMD patients. Therefore, the reader should bear in mind that results from studies on “long-term” expression in animal models might not be entirely translatable to human subjects, nor they account for the full range of muscular and systemic alterations that might be retrieved in DMD patients suffering from a longstanding disease. Having said that, current evidence can provide valuable insights on methods to enhance transgene persistence in muscle tissues.

Several lines of evidence showed that persistence of AAV genome within actively replicating, immature tissues is limited (111–113). These findings notwithstanding, some authors advocate the ability of AAV-based gene therapy to achieve therapeutic levels during early life without significant limitations to long-term expression, based on animal studies. For instance, Daly and colleagues showed that intravenous administration of an AAV encoding human beta-glucuronidase in newborn mice partially rescued the phenotype and resulted in expression up to 16 weeks of age in liver, heart, lung, spleen, kidney, brain, and retina. Notably, therapeutic levels of transgene and phenotypic rescue were not achieved in skeletal muscle (114). Other studies demonstrated persistent expression of transgene following intravenous neonatal administration in non-muscle tissues (115–118). Studies in healthy canine models yielded somewhat better results. Intravenous delivery of AAV-9-encoded micro-dystrophin in neonatal dogs led to skeletal muscle transduction throughout the body for at least 6 months, with highly transduced muscles expressing the transgene in up to 80% myofibers (94). Systemic transduction was obtained with no need of immune suppression. No study regarding intravenous administration of gene therapy for DMD has been conducted in newborn animals harboring *DMD* mutations so far.

Conversely, the IM route has been more intensively studied. IM injection of AAV during neonatal phase has previously proved able to ensure long-term expression of transgene in animal models of muscular diseases (119). IM injection of AAV-encoded micro-dystrophin in newborn dystrophic *mdx* mice allowed sarcolemmal expression of micro-dystrophin in 40 to 60% of myofibers up to 20–24 weeks of age in absence of immunosuppression in different studies (85, 87, 89). In contrast, another study showed that IM injection of an AAV encoding micro-dystrophin in *mdx* mice during the neonatal period led to high degree of short-term expression but significantly reduced long-term expression (90). Remarkably, long-term expression was restored when injection was performed in a mouse model of severe combined immunodeficiency (SCID) (90). Indeed, immune response toward the transgene seems to play a key role in impairing sustained long-term expression.

The transduction of post-mitotic or slowly replicating adult tissues should allow, at least in theory, to achieve longer genome persistence and stable transgene expression. For instance, animals treated with AAV gene transfer during adult life demonstrate persistence of vector genomes in the liver in their episomal form, with poor evidence of genomic integration, for more than 10 years (120, 121). Similarly, studies in humans proved that a single injection of AAV vector expressing human factor IX transgene led to stable transgene expression in the liver for as long as 3 years (122, 123). More recently, systemic AAV delivery of a transgene encoding micro-dystrophin was attempted in aged dystrophic *mdx* mice, with robust dystrophin expression in skeletal and/or cardiac muscle 2 to 8 months after injection (124–127). One study reported a significant immune response within the skeletal muscle of transduced mice, that was successfully prevented with steroid administration, with satisfactory therapeutic efficacy 4 weeks after transduction (128). The administration of AAV-microdystrophin via IM route in adult mice yielded successful results as well. Wang and colleagues delivered intramuscularly an AAV encoding a micro-dystrophin, expressed under the MCK promoter, in adult *mdx* mice, with a satisfactory clinical response (85). After 2 months, dystrophin was expressed by 35–50% of myofibers, while this percentage dropped to 20–30% after 4 months (85).

In diseased large mammals, body wide gene transfer has been attempted under transient or sustained immune suppression, resulting in widespread transduction in skeletal muscle, the diaphragm and heart persisting for at least 4 months (95). Evidence in wild-type canine models showed that IM injection of AAV2 or AAV6 resulted in a powerful immune response to capsid or capsid-associated proteins (93). Nonetheless, a brief course of immunosuppressive treatment was sufficient to permit long-term persistence of a canine micro-dystrophin transgene in the skeletal muscle of a DMD dog model (92). Such studies suggest that immune-mediated mechanisms might be responsible for loss of vector persistence after IM injection in big mammals such as dogs and humans, thus making the case for use of immune modulation to achieve long term transgene expression in these species. These findings notwithstanding, in 2017 an effective and safe delivery of a micro-dystrophin transgene in juvenile GRMD dog model of DMD by both intravenous locoregional and systemic administration of a rAAV2/8 vector was achieved, showing long-term persistence (up to 24 months) in the absence of preventive immunosuppression (96). However, when extending follow-up to 4 years, as performed by Elverman and colleagues in a canine model of XLMTM after loco-regional perfusion of AAV8-MTM1, a significant decline of viral copy numbers and myotubularin expression was recorded (129).

Similarly, another preclinical study led on the GRMD dog model of DMD performed a 5-year follow-up of dystrophin rescue after either IM or intravenous of AAV1-U7E6/8, an antisense sequence which induces skipping of exons 6 and 8, carried by the engineered small nuclear RNA (snRNA) U7. The authors showed an eightfold decrease of dystrophin positive myofibers, in line with the progressive loss of the U7 system (130). As no clear signs of overactivation of immune response were detected, it is possible that the decline of dystrophin expression might have resulted from the persistent pathological muscular process, which was delayed but not stopped by the truncated, not fully functional dystrophin protein (130). Intriguingly, in the exon 52-deficient *mdx* (*mdx52*) mouse model, the intravenous injection of an AAV9-U7snRNA containing an antisense sequence to skip exon 51 induced high rates of exon skipping in myofibers and cardiac fibers, but low levels of dystrophin expression, both at 8 weeks and 6 months (131). This discrepancy might originate from a deficient protein translation from the exon 51-skipped mRNA, from different mRNA levels between various DMD mouse models or from higher instability of the dystrophin protein derived from exon 51-skipped mRNA compared to others.

In addition to that, Rodino-Klapac and colleagues packaged micro-dystrophin into an AAV8 vector and delivered it via IM and intraarterial route in rhesus macaques, showing a persistent transgene expression (50–80% of myofibers after IM injection, >80% of myofibers after intra-arterial injection) for up to 3–5 months, without evidence of immune cellular infiltration, suggesting that other factors might have a role in the modulation of immune response to AAVs and transgene expression (97).

Overall, the reviewed papers point out that overactivation of the immune system represents a big challenge not only according to safety, but also in its potential to affect transgene persistence. It has been suggested that the dystrophic microenvironment might stimulate the cellular immune response, and even provoke the loss of AAV in dystrophic muscle. It seems that the use of muscle-specific synthetic promoters (e.g., MCK) might be helpful in reducing the potential for detectable cellular immune response, resulting in long-lasting transgene expression, although this surely is not the only factor implicated in the process (96, 128). Moreover, different regimens of ongoing glucocorticoid therapy (daily vs. intermittent) could influence the development of immune responses and, therefore, the long-term persistence of the transgene. Indeed, promising results were obtained in DMD patients who received a single dose of intravenous rAAVrh74.MHCK7.micro-dystrophin while being on a stable corticosteroid dose for at least 12 weeks before entry, and subsequently prednisone at high doses for 30 days, followed by a slow taper. Nonetheless, the brief follow-up does not allow to drive conclusions on this topic (60).

In this scenario, therapy re-administration over time might be required to achieve long-term efficacy but might be limited by the development of neutralizing antibodies targeting the vector. Evidence from studies conducted on other diseases might also be helpful in this regard. Based on the results of preclinical studies, a phase I/II clinical trial (NCT02240407) evaluating the re-administration of rAAV9-mediated human acid alpha-glucosidase (hGAA) in six adult patients affected by late-onset Pompe disease is currently ongoing (132). The authors provided an immune modulation approach to prevent the development of a humoral immune response against the capsid and the transgene, based on B-cell ablation with rituximab before gene therapy, whose efficacy in guaranteeing long-term persistence of transgene expression in human setting needs to be confirmed (132).

## Conclusions

AAV-mediated gene therapy is a flourishing field of discovery for human genetic and acquired disorders, including neuromuscular diseases such as DMD. After the promising results obtained by pre-clinical studies in different DMD mammalian models, scientific efforts are moving to translate these findings from the bench to the bedside. To date, several phase I/II clinical trials are ongoing, but definitive results are still lacking, thus preventing us from drawing reliable conclusions.

One critical issue associated with AAV-mediated gene therapy is the persistence of the exogenous genetic material within human cells and, more specifically, in myofibers and cardiac fibers in the case of muscular dystrophies. In addition to that, as outlined in this review of current literature, several lines of evidence suggest that enhanced host immune responses may affect transgene persistence. Indeed, a better understanding of these topics could shed light on the conundrum concerning the expected persistence of therapeutic effects following administration of gene therapy in muscular dystrophies.

Further insights from both preclinical and clinical trials are necessary also to elucidate more precisely the need for immune suppression in human subjects. So far, evidence suggests that re-administration would very likely be necessary to ensure life-long therapy in DMD patients. In this perspective, strategies to tackle the presence of preexisting neutralizing antibodies are needed, and indeed some strategies have already been proposed (e.g., use of alternative AAV capsid, immunomodulatory treatments, and plasmapheresis) (81). Further preclinical studies are ongoing to test the suitability of these approaches.

An interesting development of these molecular strategies is the potential to extend their use to mild dystrophinopathies, such as BMD (9). It has to be pointed out that most of the molecular defects in BMD concern the quantity and quality of dystrophin and are not amenable to correction with existing exon-skipping strategies, but innovative methods such as gene editing with CRISPR/Cas9 could potentially be effective (9). Further studies will be needed in this subset of patients.

In conclusion, AAV-mediated gene therapy is currently revolutionizing the world of novel therapeutic approaches for neuromuscular disorders, including DMD. The possibility to achieve long-term therapeutic effects is strongly conditioned by the persistence of transgene expression in transduced tissues, which still represents an unraveled field of investigation. Hopefully, ongoing preclinical and clinical trials will help disclose its molecular basis, and therefore overcome current obstacles in the pursuit of stable long-term outcomes.

## Author Contributions

AM, EA, and AN revised the literature and wrote the manuscript. GC conceived the idea. GC and SC performed a critical revision of the manuscript for important intellectual content. All authors have read and approved the manuscript.

## Funding

This work was supported by the Italian Ministry of Health Ricerca Corrente to GC.

## Conflict of Interest

The authors declare that the research was conducted in the absence of any commercial or financial relationships that could be construed as a potential conflict of interest.

## Publisher's Note

All claims expressed in this article are solely those of the authors and do not necessarily represent those of their affiliated organizations, or those of the publisher, the editors and the reviewers. Any product that may be evaluated in this article, or claim that may be made by its manufacturer, is not guaranteed or endorsed by the publisher.
